# Risk prediction models for malnutrition in dialysis patients in China: a systematic review and meta-analysis

**DOI:** 10.1080/0886022X.2026.2687920

**Published:** 2026-06-18

**Authors:** Mengyao Liu, Yan Wu, Fen Ye, Wenting Liu, Xu Deng, Yang Tang, Lili Deng

**Affiliations:** College of Nursing, Guangzhou University of Chinese Medicine, Guangzhou, China

**Keywords:** Dialysis patients, malnutrition, meta-analysis, risk prediction model, systematic review

## Abstract

Although multiple risk prediction models have been developed to identify malnutrition in dialysis patients, their quality and performance remain unclear, limiting their practicality in current clinical practice and future research. Therefore, we conducted a systematic review and meta-analysis to evaluate these models. Searches were conducted in PubMed, Embase, Web of Science, The Cochrane Library, CINAHL, SinoMed, CNKI, Wanfang, and VIP Database from inception to January 26, 2026. Two investigators independently screened the literature, extracted data, and assessed quality using the Prediction model Risk of Bias Assessment Tool (PROBAST). Meta-analyses of the prevalence of malnutrition, common predictors and model performance were performed using Stata 18.0 and R 4.5.1. A total of 12 eligible studies conducted in China were included, and the pooled prevalence of malnutrition in dialysis patients was 41%. Meta-analysis identified age, serum calcium, Kt/V, triglycerides, sex, vitamin D, NT-proBNP, and comorbid diabetes as statistically significant predictors. The pooled effect of the nine internal validated models was 0.83, indicating good discriminatory performance. However, all included models were rated at high risk of bias, primarily due to inappropriate data sources and poor reporting of the analysis. The current analysis reveals a high prevalence of malnutrition among dialysis patients. Eight significant predictors were identified, guiding future selection for constructing predictive models of malnutrition risk in this population. Although existing models demonstrate adequate discriminatory performance, their methodological limitations constrain clinical applicability. Future studies should prioritize the development of standardized, externally validated models to enable early identification and intervention, thereby improving outcomes in this vulnerable group.

## 1. Background

Chronic kidney disease (CKD) is a progressive condition affecting over 10% of the global population, with a total exceeding 850 million individuals [1]. When the disease progresses to end-stage renal disease (ESRD), maintenance hemodialysis and peritoneal dialysis serve as the primary renal replacement therapies [2,3], with approximately 89% of patients receiving hemodialysis and the remainder undergoing peritoneal dialysis [4]. In patients undergoing long-term dialysis, the incidence of malnutrition is significantly elevated, ranging from 18% to 75%, which is substantially higher than in the non-dialysis population [5,6]. Malnutrition in this context is primarily characterized by protein-energy wasting (PEW), a state of reduced protein and energy levels stemming from decreased nutrient intake and metabolic abnormalities [6,7]. Once it occurs, patients are at increased risk of infections, musculoskeletal deterioration, cardiovascular complications, and even mortality [8]. Therefore, early identification of dialysis patients at high risk for malnutrition is of critical clinical importance.

Given that most individual screening components have low sensitivity and specificity when used independently, effective tools for identifying patients at risk of malnutrition must incorporate multiple parameters [9]. This recognition has prompted a shift in research focus toward integrating multidimensional information to develop risk prediction models for early identification of malnutrition in dialysis patients. One study constructed multiple machine learning models using predialysis creatinine, handgrip strength, non-HDL-C, hs-CRP, and Kt/V, with the best-performing model achieving an area under the curve (AUC) of 0.924 [10]. Compared to traditional nutritional assessment tools, these multifactor integrated risk prediction models enable quantitative individual risk assessment, provide more interpretable results, and demonstrate greater potential for clinical application [11]. For instance, nomograms and intuitive digital tools developed to predict malnutrition risk in dialysis patients enable clinicians to rapidly identify high-risk individuals and determine key factors for personalized interventions, thereby significantly enhancing their clinical utility [12,13]. Consequently, the development and implementation of these models are crucial for facilitating early screening and intervention in this high-risk population.

Although risk prediction models for malnutrition in dialysis patients have been developed, significant heterogeneity persists in areas such as predictor selection, model performance, and methodological quality. In addition, the predictive accuracy and clinical applicability of these models are still unclear, which may consequently limit their clinical application. However, no systematic review has comprehensively evaluated these malnutrition risk prediction models for dialysis patients. Therefore, this study aimed to conduct a systematic review to rigorously assess the methodological quality and clinical utility of existing models and summarize the key predictive factors thereof. In addition, we sought to estimate the prevalence of malnutrition in this population, thereby providing evidence for informing clinical practice and guiding future research.

## 2. Methods

This study was reported in accordance with the Preferred Reporting Items for Systematic Reviews and Meta-Analyses (PRISMA) guidelines [14]. The study protocol was registered with PROSPERO (registration number: CRD42025631593).

### 2.1. Search strategy

We conducted a comprehensive search of both Chinese and English databases, including PubMed, Embase, Web of Science, The Cochrane Library, CINAHL, Chinese Biomedical Literature Database (SinoMed), China Science and Technology Journal Database (VIP), Wanfang, and China National Knowledge Infrastructure (CNKI). The search spanned from each database’s inception to January 26, 2026. Search strategies were developed using Medical Subject Headings (MeSH) combining free terms, including ‘Malnutrition’, ‘Protein-Energy Malnutrition’, ‘Dialysis’, ‘Hemodialysis’, ‘Peritoneal dialysis’, ‘Risk prediction’, ‘Predictive model’, ‘Nomogram’. Detailed search strategies are provided in Table S1. Furthermore, we adopted the PICOTS system (Table 1), which is recommended by the CHARMS checklist [15]. This system facilitated the definition of the review’s objective, and guided the development of search strategies, literature screening, data extraction, and analysis.

**Table 1.** PICOTS framework for study selection.

Item	Definition
Population	Patients diagnosed with end-stage renal disease who undergo peritoneal dialysis and/or hemodialysis, and are aged ≥ 18 years
Intervention	Development and/or external validation of risk prediction models for malnutrition in dialysis patients
Comparator	Not applicable
Outcome	Malnutrition during dialysis
Timing	Using the predictor variables identified through the screening process
Setting	Hospitals or hemodialysis centers

### 2.2. Inclusion and exclusion criteria

Inclusion criteria: (1) Target population: Patients with end-stage renal disease undergoing peritoneal dialysis and/or hemodialysis; (2) Study design: observational studies, such as cross-sectional studies, cohort studies, and case-control studies; (3) Type of prediction model: risk prediction models developed or externally validated existing models using statistical methods, with model performance evaluated by at least one indicator; (4) Outcome: Malnutrition, as defined using valid malnutrition assessment tools.

Exclusion criteria: (1) Study protocols, reviews, conference abstracts, letters, and comments; (2) Studies that only analyze risk factors without developing or validating prediction models; (3) Studies not written in English or Chinese.

### 2.3. Study selection

Two researchers independently conducted the study selection process in three sequential phases. First, all search results were imported into Endnote 21 to detect and remove duplicate studies. Second, irrelevant studies were excluded through screening titles and abstracts based on the pre-defined inclusion and exclusion criteria. Third, full-text articles of potentially eligible studies were assessed, and their reference lists were manually searched to identify additional relevant publications. Any discrepancies between reviewers were resolved through discussion with a third researcher to reach consensus.

### 2.4. Data extraction

Two researchers independently developed a standardized data collection form based on the CHARMS [15], and subsequently performed data extraction for the included studies. The extracted information comprised: (1) Study characteristics: first author, study design, data source, participants, dialysis method, outcome measures and sample size. (2) Model characteristics: development methodology, continuous variable processing method, model validation method, model presentation form, predictive factors. Should disagreements arise during data extraction, a third researcher was consulted to reach consensus.

### 2.5. Quality assessment

Two researchers independently performed quality assessment of the included studies. Any discrepancies were resolved through discussion with a third researcher until consensus was reached. The Grading of Recommendations Assessment, Development, and Evaluation (GRADE) [16] and Prediction Model Risk of Bias Assessment Tool (PROBAST) [17] were used independently to evaluate the risk of bias and quality. GRADE comprehensively assesses the quality of evidence through six dimensions, including study design, risk of bias, inconsistency, indirectness, imprecision, and publication bias. The overall level of evidence is categorized into four levels: high, moderate, low, and very low. PROBAST evaluates prediction model studies across four domains, including participants, predictors, outcomes, analysis. It comprises 20 questions to evaluate risk of bias in design, conduct, and analysis. Each question is rated as ‘Yes/Probably yes’, ‘Probably no/No’, or ‘No information’. A model is considered at low overall bias risk only if all domains are rated as low risk, at high risk if any domain is rated high, and at unclear risk if any domain is unclear while others are low. Applicability is evaluated through three domains (participants, predictors, outcomes) and rated as low, high, or unclear risk. Overall applicability is considered good if all domains are low risk and poor if any domain is high risk.

### 2.6. Statistical analysis

Statistical analysis was performed using Stata 18.0 and R 4.5.1. The pooled prevalence of malnutrition among dialysis patients was estimated through a meta-analysis of single-group rates, with results presented as the overall prevalence and its 95% confidence interval [18]. For predictive factors extracted from the models, odds ratios (*ORs*) with *95% CIs* were used as effect measures. Model discrimination was evaluated by pooling the AUC. Heterogeneity was quantified using the *I^2^* statistic; a fixed-effect model was applied when *p* > 0.1 and *I*^2^ < 50%, and a random-effects model was used otherwise [19]. To explore sources of heterogeneity, sensitivity and subgroup analyses were conducted. Publication bias was assessed using funnel plots and Egger’s regression test, with *p* > 0.05 indicating a low likelihood of bias. If significant publication bias was detected, the trim-and-fill method was applied to adjust for potentially missing studies and to evaluate the robustness of the pooled estimates [20].

## 3. Results

### 3.1. Search results

**Figure 1.**
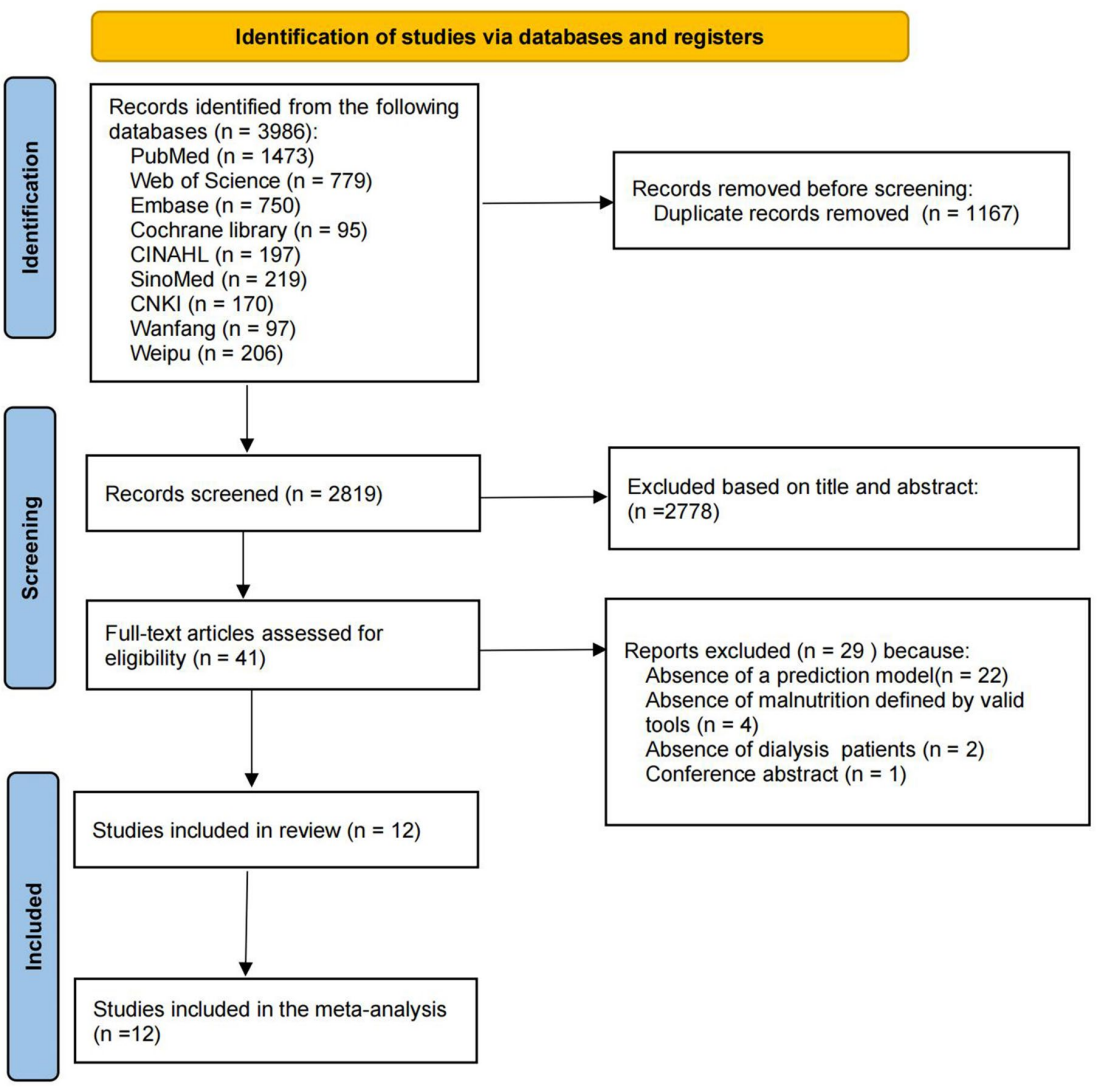
PRISMA 2020 flow diagram of literature search and selection.

The study selection process is shown in Figure 1. A total of 3,986 records were identified through the search strategy, of which 1,167 duplicates were removed. After reviewing the titles and abstracts, 2,778 irrelevant studies were excluded. Full-text screening was performed for the remaining 41 records. Subsequently, 29 studies were excluded: 22 studies that did not develop prediction models or only analyzed risk factors, four that did not use validated assessment tools to define malnutrition, two studies with a target population inconsistent with this review, and one study published as a conference abstract. Ultimately, 12 studies were included in this review.

### 3.2. Characteristics of included studies

Table 2 comprehensively summarizes the study characteristics of the 12 included studies. These studies were published in China between 2022 and 2025. Of these included studies, five were cross-sectional studies (including three multicenter studies) and seven were retrospective studies (including one multicenter study). In terms of dialysis modality, nine studies involved hemodialysis populations, two involved peritoneal dialysis populations, and one included both. For the assessment tools for malnutrition, the Protein-Energy Wasting (PEW) was most commonly used (*n* = 7) [7]. Other assessment tools were the Modified Quantitative Subjective Global Assessment (*n* = 2) [22], Controlling Nutritional Status (*n* = 1) [23], Subjective Global Assessment (*n* = 1) [24], and Mini Nutritional Assessment Short-Form (*n* = 1). The sample size of each study ranged from 126 to 1355 cases. The incidence of malnutrition in each study ranged from 21.42% to 54.25%.

**Table 2.** Characteristics of included studies (*n* = 12).

Author (year)	Region (Province)	Study design	Data source	Participants	Study population	Outcome measures	Sample Size			Malnutrition cases/sample size (%)
							D	I	E	
Min Wei^a^ [25]	Eastern (Jiangsu)	Retrospective study	One hospital (December 2019-December 2021)	Aged ≥18 years and with maintenance hemodialysis duration ≥6 months.	HD	SGA	153	102	/	83/153 (54.25%)
Guoting Ma^a^ [26]	Western (Sichuan)	Retrospective study	Six hemodialysis centers (November 2021-May 2022)	Aged ≥18 years and with maintenance hemodialysis duration ≥3 months.	HD	CONUT	1355	/	/	325/1355 (23.99%)
Xueqin Liu^a^ [13]	Central (Henan)	cross-sectional	Three hospitals (March-October 2022)	Maintenance hemodialysis duration ≥3 months.	HD	MQSGA	320	/	137	234/457 (51.20%)
Weina Wang [27]	Southern (Zhejiang)	cross-sectional	Two Hospital (October 2018–September 2020)	Aged 18–80 years and receiving regular peritoneal dialysis or hemodialysis for ≥ 3 months.	PD and HD	PEW	448	161	52	109/448 (24.33%)
Ziwei Mei^a^ [28]	Southern (Zhejiang)	Retrospective study	Two Hospital (January 2011- August 2022)	Aged 18–80 years and Peritoneal dialysis duration ≥3 months.	PD	PEW	211	77	/	125/288 (43.40%)
Si Chen [29]	Southern (Shanghai)	cross-sectional	Four hospitals	Aged 18–75 years and with maintenance hemodialysis duration ≥6 months.	HD	PEW	380	/	/	175/380 (46.05%)
Danying Yan [30]	Southern (Shanghai )	cross-sectional	Five hospitals	Aged 18–75 years and with maintenance hemodialysis duration ≥6 months.	HD	PEW	/	/	622	287/622 (46.14%)
Ziwei Mei [12]	Southern (Zhejiang)	Retrospective study	Two hospitals (January 2011-November 2022)	Aged >18 years; Peritoneal dialysis duration ≥3 months.	PD	PEW	210	159	/	184/369 (49.86%)
Mingmei Ding^a^ [31]	Eastern (Jiangsu)	Retrospective study	One hemodialysis center (February 2020- February 2023)	Aged 18–75 years and with maintenance hemodialysis duration ≥6 months.	HD	PEW	313	/	/	125/313 (39.94%)
Han Zhao^a^ [32]	Eastern (Jiangsu)	Retrospective study	One hospital (July 2018– August 2023)	Aged 20-65 years and with maintenance hemodialysis duration ≥3 months.	HD	MNA-SF	150	/		62/150 (41.33%)
Genlian Cai [10]	Southern (Shanghai)	cross-sectional study	One medical institution (January 2024 to June 2024)	Aged ≥18 years and with maintenance hemodialysis duration ≥6 months.	HD	PEW	635	273		136/635 (21.42%)
Yingying Zhang^a^ [33]	Southern (Anhui)	Retrospective study	One hospital (January 2022– June 2024)	Aged ≥18 years and with maintenance hemodialysis duration ≥12 weeks.	HD	MQSGA	126	/	/	67/126 (53.17%)

Note: ‘/’, not reported. ‘a’, Study was published in Chinese. D, Model development; I, Internal validation; E, External validation; PEW, Protein-Energy Wasting; HD, Hemodialysis.

PD, Peritoneal Dialysis; SGA, Subjective Global Assessment; CONUT, Controlling Nutritional Status.

MQSGA, Modified Quantitative Subjective Global Assessment; MNA-SF, Mini Nutritional Assessment Short-Form.

### 3.3. Model characteristics of included studies

Table 3 systematically summarizes the model characteristics of included studies. The majority of studies utilized multivariable logistic regression for model development, with only one employing a machine learning approach. Most included models underwent internal validation, with only three studies performing external validation. The AUC was the most widely used discrimination metric, with values ranging from 0.764 to 0.994 during development, from 0.663 to 0.985 in internal validation, and from 0.777 to 0.875 in external validation. Nomograms were the most common presentation format. Calibration was reported for 11 models, most commonly assessed using the Hosmer–Lemeshow test. The number of predictive factors varied from 4 to 11, and the most frequently included predictors comprised age (*n* = 7), Kt/V (*n* = 5), triglycerides (*n* = 4), serum calcium (*n* = 3), and dialysis duration (*n* = 3).

**Table 3.** Model characteristics of included studies. (*n* = 12).

Author (year)	Development methodology	Continuous variable	Validation Methodology	AUC (95% CI)			Other Performance	Model Presentation	Calibration method	Predictors
				D	I	E				
Min Wei [25]	Multivariate logistic regression	Continuous Categorical	Internal Validation	0.877	0.825	/	D,Sensitivity = 84.34%, Specificity = 80.00%; I,Sensitivity = 89.09%, Specificity = 61. 70%	Nomogram	Hosmer-Lemeshow	Insufficient protein energy intake, Kt/V, Age, Dialysis duration, Hs-CRP
Guo ting Ma [26]	Multivariate logistic regression	Categorical	Internal Validation (bootstrap)	/	0.862	/	I,Sensitivity = 86.20% Specificity = 72.80%	risk assessment graph	/	Age ≥ 60, Comorbid diabetes, Kt/V, Intradialytic hypotension within past 1 month, PTH
Xueqin Liu [13]	Multivariate logistic regression	Continuous, Categorical	External validation	0.881	/	0.875	D,Sensitivity = 89.20%, Specificity = 77.80% E,Sensitivity = 80.00%, Specificity = 84.70%	Nomogram, online calculator	Hosmer-Lemeshow	Age ≥ 60, Dialysis duration, Kt/V, ALB, Hb, Depression score
Weina Wang [27]	Multivariate logistic regression	Continuous	Internal Validation, External Validation (bootstrap)	0.843	0.841	0.829	I,Sensitivity = 73.20% Specificity = 78.30%	Nomogram, Risk score	Hosmer-Lemeshow	Age, Dialysis modality, SGA, Triglyceride, Urea nitrogen, Calcium, Ferritin, BCM, Water ratio, VFA, Phase angle
Ziwei Mei [28]	Multivariate logistic regression	Continuous	Internal Validation	0.764	0. 663	/	/	Nomogram	Hosmer-Lemeshow	Age, Serum calcium, Serum sodium, Fasting blood sugar, Urea clearance index, Ultrafiltration volume
Si Chen [29]	Multivariate logistic regression	Continuous	Internal Validation (bootstrap)	/	0.851	/	/	Nomogram	Calibration curve	BMI, ALB, TC,Sex, TG, Vit D, NT-proBNP
Danying Yan [30]	/	Continuous	External validation	/	/	0.777	C-index: 0.777	Nomogram	Brier score	BMI, ALB, TC, Sex, TG, Vitamin D, NT-proBNP
Ziwei Mei [12]	Multivariate logistic regression	Continuous	Internal Validation	0.769	0.669	/	/	Nomogram	Calibration plots	Scr, Age, Ccr, Dialysis duration, TG, Serum calcium, Glucose, CRP
Mingmei Ding [31]	Multivariate logistic regression	Continuous	Internal Validation (bootstrap)	/	0.867	/	C-index: 0.875	Nomogram	Hosmer-Lemeshow	Sex, NT-pro BNP, Vitamin D, TC
Han Zhao [32]	Multivariate logistic regression	Continuous	Internal Validation (bootstrap)	0.994	0.985	/	C-index: 0.994	Nomogram	Calibration curve	MAC, MAMC, Hb, social support rating scale score, self-efficacy questionnaire score
Genlian Cai [10]	Lasso regression analysis	Continuous	Internal Validation	0.924	0.827	/	D,Sensitivity = 88.20%, Specificity = 83.40%; I,Sensitivity = 72.70%, Specificity = 76.20%	Web application	calibration curve and decision curve analysis curve	Creatinine, handgrip, non-HDL-C, hs-CRP, Kt/V
Yingying Zhang [33]	Multivariate logistic regression	categorical	/	0. 829			/	Nomogram	Hosmer-Lemeshow	Age, Comorbid diabetes, Concurrent renal anemia, Kt/V

Note: ‘/’, not reported; D, Model development; I, Internal validation; E, External validation; AUC, Area Under the Curve; Kt/V, Kt/V urea; Hs-CRP, High-sensitivity C-reactive protein; PTH, Parathyroid Hormone; ALB, Albumin; Hb, Hemoglobin; SGA, Subjective Global Assessment; TG, Triglyceride; BCM, Body Cell Mass; VFA, Visceral Fat Area; BMI, Body Mass Index; TC, Total Cholesterol; Ccr, Creatinine Clearance Rate; CRP, C-reactive protein; MAC, Mid-upper arm circumference; MAMC, Mid-upper arm muscle circumference.

### 3.4. Results of quality assessment

The GRADE evidence profile for the pooled review’s effect was graded as moderate, mainly due to inconsistencies in outcome definition across studies, which contribute to imprecision in the pooled estimate (Table S2).

Table 4 summarizes the risk of bias and applicability assessments for the 12 included studies, with all studies having a high risk of bias. In the participant domain, seven studies had a high risk of bias primarily due to inappropriate data sources [12,25, 26,28, 31–33]. Their data sources came from retrospective study design. In the predictor domain, six studies had unclear risk of bias primarily due to not reporting information on blinding of predictor assessment to outcome data [12,25, 28,31–33]. In the outcome domain, three studies had a high risk of bias primarily due to the predictors used by the model being included in the definition of the outcome [13,29,30]. In the analysis domain, all studies had a high risk of bias. Among them, four studies had insufficient sample sizes, failing to meet the assessment requirement of ≥ 20 events per predictor variable (EPV) [25,28, 32,33]. Four studies categorized continuous predictor variables into ≥ 2 categories [13,25, 26,33]. One study did not comprehensively assess the discrimination and calibration of the predictive models [26]. Four studies did not consider overfitting, underfitting, and optimal fitting of the prediction model [12,13, 25,28]. Only one study mentioned the method used to handle missing data [10]. All studies ignored information on data complexities. For the applicability assessment, one study was rated as high risk, primarily due to age restrictions on the study subjects [32].

**Table 4.** PROBAST results of the included studies. (*n* = 12).

Study	ROB				Applicability			Overall	
	Participants	Predictors	Outcome	Analysis	Participants	Predictors	Outcome	ROB	Applicability
Min Wei [25]	−	?	+	−	+	+	+	−	+
Guoting Ma [26]	−	+	+	−	+	+	+	−	+
Xueqin Liu [13]	+	+	−	−	+	+	+	−	+
Weina Wang [27]	+	+	+	−	+	+	+	−	+
Ziwei Mei [28]	−	?	+	−	+	+	+	−	+
Si Chen [29]	+	+	−	−	+	+	+	−	+
Danying Yan [30]	+	+	−	−	+	+	+	−	+
Ziwei Mei [12]	−	?	+	−	+	+	+	−	+
Mingmei Ding [31]	−	?	+	−	+	+	+	−	+
Han Zhao [32]	−	?	+	−	−	+	+	−	−
Genlian Cai [10]	+	+	+	−	+	+	+	−	+
Yingying Zhang [33]	−	?	+	−	+	+	+	−	+

PROBAST, Prediction model Risk of Bias Assessment Tool; ROB, risk of bias.

+ indicates low ROB/low concern regarding applicability.

− indicates high ROB/high concern regarding application.

? indicates unclear ROB/unclear concern regarding applicability.

### 3.5. Meta-analysis findings

#### 3.5.1. Prevalence of malnutrition in dialysis patients

**Figure 2.**
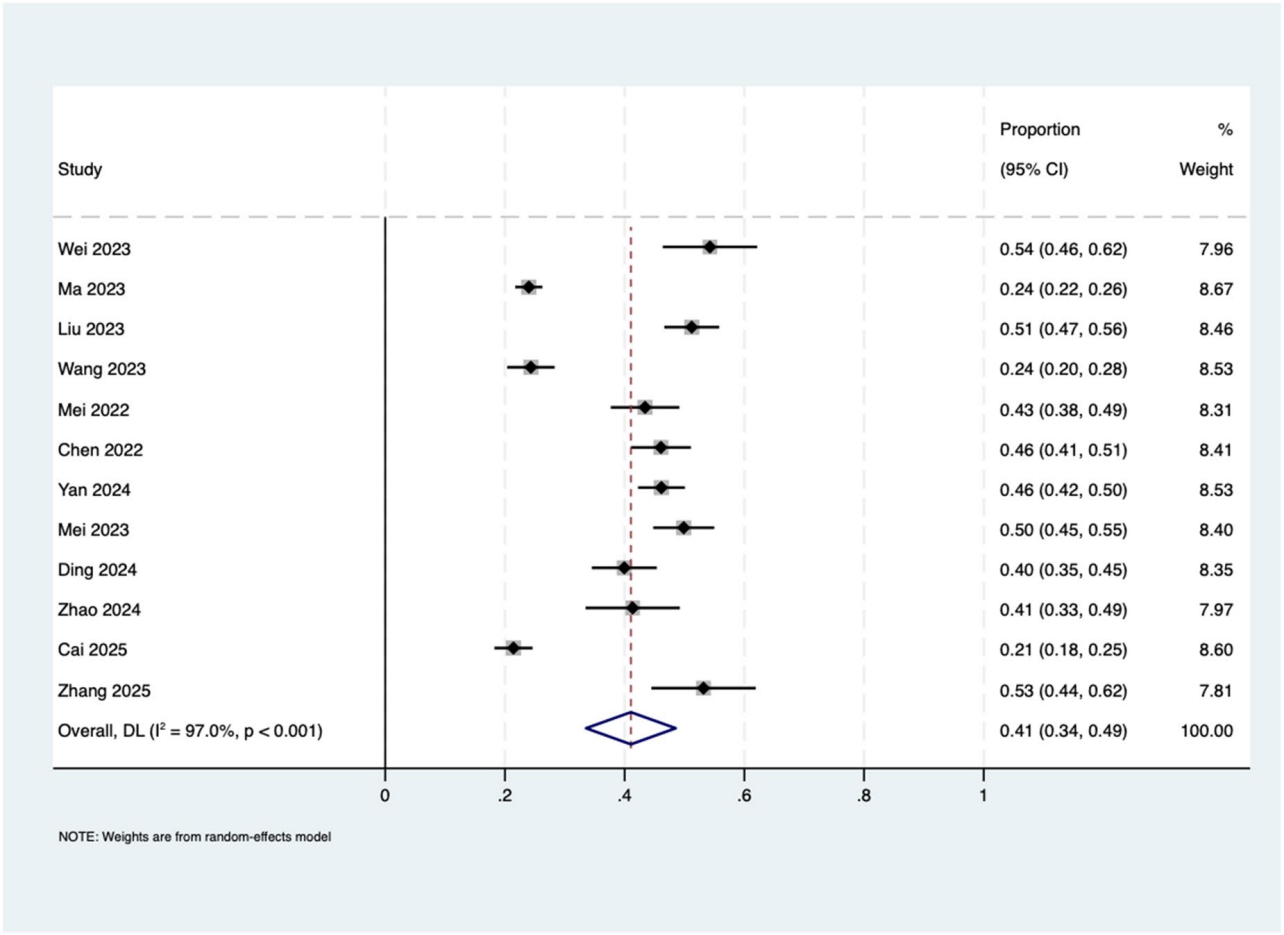
Forest plot of the prevalence of malnutrition in dialysis patients.

A meta-analysis of data from all included studies using a random-effects model showed that the overall prevalence of malnutrition in dialysis patients was 41% (*95% CI*: 0.34-0.49), with substantial heterogeneity (*I*^2^ = 97.0%, *p* < 0.001) (Figure 2). To explore potential sources of heterogeneity, subgroup analyses were conducted. These analyses revealed that dialysis modality and nutritional assessment tools were the primary sources of heterogeneity (*p* < 0.001) (Figure S1).

#### 3.5.2. Predictors for malnutrition in dialysis patients

As shown in Table 5 and the forest plot in Figure S2, a meta-analysis was performed on common predictors reported in at least two studies. 10 studies were included for pooled analysis, while two studies without independent effect estimates were excluded. The results indicated that age, serum calcium, Kt/V, triglycerides, sex, vitamin D, NT-proBNP, and comorbid diabetes were significantly associated with the prevalence of malnutrition (*p* < 0.05). In contrast, dialysis duration and hemoglobin showed no statistically significant associations in the meta-analysis. Since optimal cutoff values were not reported in the included studies, the baseline characteristics of these predictors are presented in Table S3.

**Table 5.** Meta-analysis results of common predictors of the model.

Number of study	Predict factors	Heterogeneity test		Effect model	Effect size combination			
		I2	*p* value		Z	OR	95%	*p*
7	Age	94.9%	<0.001	Random	3.86	1.21	1.10-1.33	<0.001
4	Kt/V < 1.2	0.0%	0.477	Fixed	8.01	3.43	2.53-4.63	<0.001
3	Dialysis duration	93.6%	<0.001	Random	0.89	1.10	0.89-1.35	0.375
3	Serum calcium	25.4%	0.262	Fixed	−5.09	0.12	0.06-0.28	<0.001
3	Triglycerides	20.4%	0.285	Fixed	−4.21	0.80	0.73-0.89	<0.001
2	Sex	34.6%	0.216	Fixed	2.86	1.25	1.07-1.46	0.004
2	Vitamin D	0.0%	0.578	Fixed	−3.87	0.87	0.82-0.94	<0.001
2	Hemoglobin	97.5%	0.000	Random	0.59	1.03	0.93-1.15	0.555
2	NT-proBNP	23.7%	0.252	Fixed	2.55	1.08	1.02-1.14	0.011
2	Comorbid diabetes	36.6%	0.209	Fixed	5.42	2.60	1.84-3.67	<0.001

For age with high heterogeneity, leave-one-out sensitivity analysis showed no obvious decline in heterogeneity after omitting any single study. Subgroup analysis indicated that inconsistent handling of the age variable was the main source of inter-study heterogeneity (Figure S3).

#### 3.5.3. Meta-analysis of predictive model effect sizes

**Figure 3.**
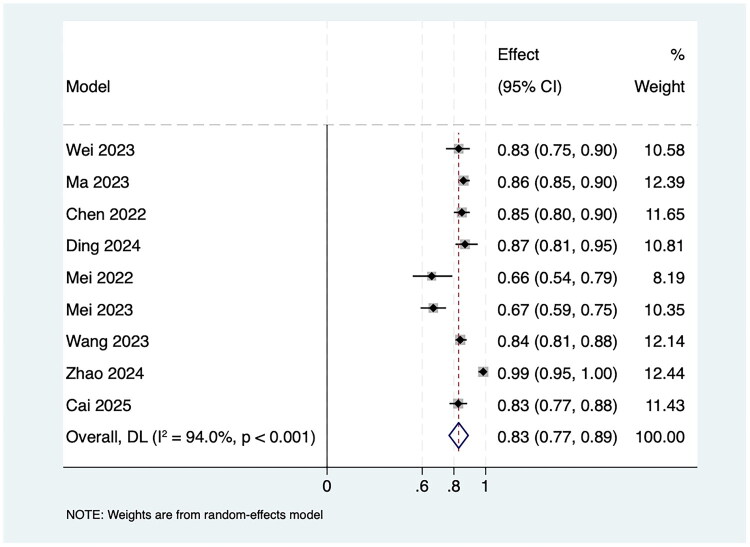
Forest plot of the pooled AUC of nine prediction models.

**Figure 4.**
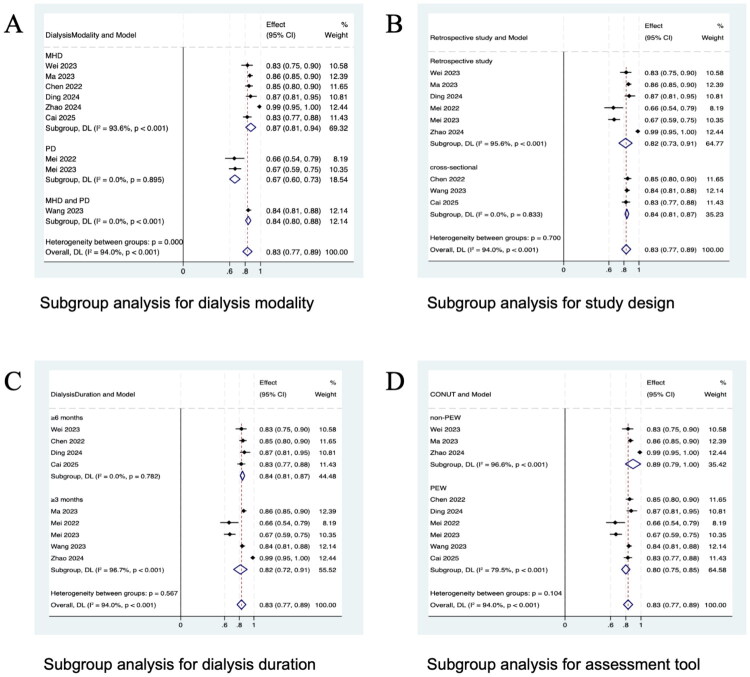
Subgroup analysis using pooled AUC to explore potential sources of between-study heterogeneity.

Due to inadequate reporting of the model effect sizes in the included studies, only nine studies were eligible for synthesis. Meta-analysis of internal validation AUC values from these models yielded a pooled AUC of 0.83 (*95% CI:* 0.77-0.89), with substantial heterogeneity (*I*^2^ = 94.0%, *p* < 0.001) (Figure 3). Subgroup analyses were conducted based on four dimensions: study design, dialysis modality, dialysis duration, and malnutrition assessment tool. Results showed that dialysis modality was identified as a key factor contributing to the heterogeneity, with statistically significant between-group differences (*p* < 0.001). The effect sizes were 0.87 (*95% CI*: 0.81-0.94) for hemodialysis and 0.67 (*95% CI*: 0.60-0.73) for peritoneal dialysis (Figure 4). Publication bias was assessed using Egger’s test, which yielded a value of 3.113 (*p* = 0.035), suggesting possible publication bias among the included studies (Figure S4). A trim-and-fill analysis was conducted incorporating nine studies to evaluate robustness. The pooled estimate remained stable, indicating that the conclusions were not substantially affected (Figure S5).

## 4. Discussion

Early diagnosis of malnutrition is critical for improving prognosis in dialysis patients [34]. As a preventable and modifiable complication, timely identification creates a crucial therapeutic window for clinical intervention, significantly improving quality of life and reducing complication risks [35,36]. The importance of developing risk prediction models for malnutrition is receiving growing attention. This systematic review identified 12 relevant studies. The pooled analysis revealed a high prevalence of malnutrition risk in this population, at 41% (*95% CI*: 0.34–0.49). Several factors were confirmed as effective predictors, including age, serum calcium, Kt/V, triglycerides, sex, vitamin D, NT-proBNP, and comorbid diabetes. Meta-analysis of nine validated models demonstrated good discriminatory ability, with a pooled AUC of 0.83 (*95% CI*: 0.77–0.89). However, according to the PROBAST assessment tool, all studies were considered to have a high risk of bias, primarily due to inappropriate data sources and poor reporting of the analysis. These methodological limitations may constrain the clinical utility of the existing models. Therefore, future research should focus on refining current models and developing new ones with improved methodological rigor.

### 4.1. Analysis of malnutrition prevalence variations for dialysis patients

The included studies showed significant variations in malnutrition prevalence among dialysis patients. Subgroup analysis indicated that this heterogeneity was due to variations in dialysis modality and nutritional assessment tools. First, the risk of malnutrition differs between dialysis modalities due to their inherent physiological differences [37,38]. For hemodialysis, the increased risk of malnutrition is associated with greater nutrient loss, a persistent state of micro-inflammation, and the need for stricter dietary restrictions [39]. For peritoneal dialysis, the risk of malnutrition is from the material transport process between the dialysis solution and the peritoneum, which increases protein loss and thus elevates the risk of malnutrition [40]. Second, this review identified several assessment tools utilized to evaluate outcomes in malnutrition risk prediction models. Among these, the criteria for PEW were the most frequently employed. These tools exhibited marked heterogeneity in their sensitivity, specificity, and diagnostic criteria. A large meta-analysis confirmed that the reported prevalence of malnutrition in hemodialysis patients can vary from 28% to 56% solely due to the use of different assessment tools, highlighting the critical importance of tool standardization [21]. Therefore, to improve the comparability and generalizability of future risk prediction models, research should be conducted in well-defined, homogeneous dialysis populations using standardized assessment tools.

### 4.2. Analysis of predictors in malnutrition risk prediction models for dialysis patients

Meta-analysis identified several statistically significant predictors of malnutrition, including advanced age, sex, serum calcium, Kt/V, triglycerides, vitamin D, NT-proBNP, and comorbid diabetes. These findings provide a foundation for healthcare professionals to implement early risk screening. Elderly dialysis patients are more susceptible to sarcopenia and nutritional deterioration due to age-related decline in organ function coupled with the metabolic burden of dialysis itself [41]. Female patients, influenced by physiological traits, metabolic changes, and dietary behaviors, face a significantly elevated risk of malnutrition [42]. The presence of diabetes further complicates the condition by promoting muscle catabolism and worsening metabolic disturbances through insulin resistance. This necessitates a careful balance between stringent glycemic control and adequate nutritional support. [43,44]. This highlights the importance of prioritizing elderly, female, and diabetic patients through structured nutritional assessment protocols and individualized intervention strategies.

Although Kt/*V* < 1.2 was a critical variable associated with malnutrition in this meta-analysis, this finding should be interpreted with caution, partly because Kt/V is influenced by the volume of distribution (V) [45,46]. For instance, the normalized protein catabolic rate (nPCR) – which is a marker of nutritional status – shares V in its calculation with Kt/V, which may artificially inflate their correlation [47,48]. Therefore, when developing predictive models or in clinical practice, patient assessment should not rely solely on Kt/V as a standalone marker. Instead, it would be better to interpret Kt/V alongside other nutritional, biochemical, and clinical indicators to facilitate a comprehensive assessment of malnutrition in dialysis patients.

Regarding biochemical monitoring, vitamin D sufficiency was a protective factor in this meta-analysis. However, owing to impaired renal activation, dietary restrictions, and urinary loss of vitamin D-binding protein, vitamin D deficiency (< 20 ng/mL) is highly prevalent in this population. This deficiency not only compromises patients’ nutritional status but also increases mortality risk [49]. NT-proBNP was identified as a significant risk factor for malnutrition in dialysis patients, with higher levels observed in the malnutrition group across all included studies. It may increase the risk of malnutrition by mediating chronic inflammation and exacerbating pathological processes such as fluid overload and myocardial injury [50]. However, routine monitoring of these biomarkers has not been widely implemented across dialysis centers, potentially limiting opportunities for early risk identification and intervention. Given this, further consideration could be given to incorporating vitamin D and NT-proBNP into periodic assessment protocols, as this could help develop a more comprehensive framework for monitoring nutritional risk.

High level of serum calcium was identified as a robust protective factor in this meta-analysis, and consistently lower serum calcium levels were observed in the malnutrition group across all included studies. This phenomenon may be attributable to dialysis-related dietary restrictions, reduced gastrointestinal calcium absorption, and impaired calcium transport [51]. Long-term hypocalcemia (< 2.10 mmol/L) can trigger secondary hyperparathyroidism, thereby inducing renal osteodystrophy and further deteriorating nutritional status [52]. Triglycerides serve as an important form of energy reserve in dialysis patients and can reflect their nutritional status to a certain extent [53]. In this meta-analysis, elevated triglyceride levels were identified as a protective factor, with significantly lower levels observed in the malnutrition group. These findings suggest that triglyceride levels in dialysis patients should not be reduced too aggressively, as this may adversely affect nutritional status. However, nutritional assessment requires a combination of multiple indicators, including anthropometric measurements and serum albumin, rather than relying merely on a single biochemical marker to avoid one-sided conclusions.

Notably, all included studies only reported odds ratios (ORs) for the associations between these biochemical markers and malnutrition, without exploring dose–response relationships or specific threshold effects. Future predictive models should establish optimal cutoff values or clinical reference ranges linked to patient outcomes to improve clinical interpretability and practical applicability.

Physical function is a core dimension of nutritional assessment [5], yet it is frequently overlooked in existing malnutrition risk prediction models for dialysis patients. Among the included studies, only one incorporated handgrip strength as a predictor [10], despite its established validity as an indicator of nutritional status in this population [54,55]; future studies should consider including such physiological function indicators alongside key clinical variables to develop more comprehensive and accurate prediction models. Although dialysis duration is a recognized predictor and is clearly associated with malnutrition in dialysis patients [56,57], this study did not identify it as an independent significant risk factor. This may be due to the small number of included original studies (only three) and the substantial heterogeneity across studies. Given the limited number of available studies and insufficient sample sizes, large-scale clinical data are still needed to further validate these findings, and the meta-analytic estimates for these variables should be interpreted with caution.

### 4.3. Analysis of performance and bias risk in malnutrition risk prediction models for dialysis patients

During the systematic review of 12 existing malnutrition risk prediction models for dialysis patients, we observed AUC values ranging from 0.663 to 0.994, of which 10 demonstrated an AUC exceeding 0.8, indicating good to excellent predictive ability. Meta-analysis yielded a pooled AUC of 0.83 (*95% CI*: 0.77-0.89), further confirming the overall reliability of the current models in predicting malnutrition risk in dialysis patients. However, the PROBAST assessment revealed a high risk of bias across all studies, substantially limiting their potential for clinical application.

For participants domain, a high risk of bias was identified, primarily due to the use of retrospectively collected data in seven studies [12,25,26,28,31–33]. As these data were not originally intended for model development or validation, they are susceptible to biases such as recall bias, missing data, and selective reporting. Future studies should prioritize prospective cohort or nested case-control designs to reduce such bias. For predictors, six studies did not report whether blinding was used during outcome determination and predictive factor measurement [12,25,28,31–33]. This omission may increase the risk of incorporating outcome information into predictor evaluation, potentially inflating the observed associations and leading to overestimated model performance. For outcome, three studies included predictors of malnutrition that were also included in the outcome definition [13,29,30], which may lead to incorporation bias by artificially overestimating the association between predictors and the outcome.

In the analysis domain, several methodological limitations were identified. First, four studies had insufficient sample sizes, which increased the risk of model overfitting and may have biased the estimation of predictive performance [25,28,32,33]. Future studies should perform a priori sample size calculations to ensure the accuracy and robustness of model parameters [58]. Second, four studies inappropriately categorized continuous variables, which can reduce predictive accuracy and attenuate the correlation between predictors and outcomes [13,25,26,33]. Future studies should maintain continuous variables in their original scale or apply predefined, clinically justified cutoffs when categorization is necessary. Third, with the exception of one study, the handling of missing data was not clearly reported [10], compromising the transparency and potentially introducing bias into predictor-outcome association estimates. Fourth, one study failed to assess both calibration and discrimination, thereby limiting the evaluation of the model’s ability to provide accurate individual probability estimates and potentially introducing bias [26]. Additionally, four studies applied internal validation methods incorrectly, which may lead to overfitting or underfitting [12,13, 25,28]. In conclusion, future research should adhere to the Transparent Reporting of a Multivariable Prediction Model for Individual Prognosis or Diagnosis (TRIPOD) guidelines, with particular attention to sample size estimation, appropriate variable handling, and rigorous validation during the study design phase, alongside transparent reporting of all analytical procedures [59]. Employing quality assessment tools such as PROBAST early in the research process can help identify and address methodological weaknesses, thereby enhancing the scientific validity and clinical applicability of malnutrition risk prediction models for dialysis patients [17].

Currently, most research on malnutrition prediction models for dialysis patients is still in the development and internal validation stage, with only three studies having performed external validation [13,27,30]. Insufficient emphasis on external validation has raised concerns regarding the generalizability of these models, which directly impedes their effective translation into clinical practice. Consequently, future efforts in developing such prediction models should prioritize external validation through multicenter, large-sample studies to ensure their reliability and applicability in real-world settings. Additionally, the majority of malnutrition prediction models continue to rely on logistic regression, with only one study having employed machine learning methods. The strength of logistic regression lies in its high interpretability and straightforward translation into clinical scoring tools or nomograms, facilitating its adoption in clinical practice [60]. However, as the dimensionality and complexity of clinical data grow, the capacity of logistic regression to process such data may become limited [61]. In contrast, machine learning and artificial intelligence techniques have attracted increasing interest due to their ability to handle high-dimensional and nonlinear data, offering novel opportunities for more precise risk stratification [62]. Future research should expand the exploration of these advanced methodologies, including the integration of traditional statistical and machine learning approaches, to develop models with enhanced predictive performance.

### 4.4. Limitations and implications for future research

The principal strength of the present study is that it represents the first systematic review and meta-analysis of malnutrition risk prediction models in dialysis patients, providing a comprehensive evaluation of the methodological quality, predictive performance, and clinical applicability of existing models, as well as identifying and analyzing the key factors influencing malnutrition in this population to support clinical risk identification. However, several limitations should be acknowledged: (1) the data used to develop the included models were predominantly derived from Chinese populations, which limits the geographical generalizability of the findings and their applicability to Western settings. Future research should incorporate data from multiple countries to enhance the general applicability of the models; (2) only Chinese and English publications were included, which may introduce language bias and lead to the omission of relevant studies published in other languages. Multilingual literature searches are recommended in subsequent reviews; (3) this review combined models developed for hemodialysis and peritoneal dialysis patients. Given the distinct pathophysiological mechanisms and clinical management between these modalities, this approach may have increased heterogeneity. Future systematic reviews should consider analyzing these groups separately; (4) due to the limited number of studies, meta-analysis was performed only for predictors reported in two or more studies, which may have resulted in incomplete synthesis of potentially important factors. Further validation is needed as more studies accumulate in the future.

## 5. Conclusion

Existing research indicates that malnutrition is highly prevalent among dialysis patients. Age, serum calcium, Kt/V, triglycerides, sex, vitamin D, NT-proBNP, and comorbid diabetes are identified as significant predictors of malnutrition in this population. In clinical practice, greater attention should be paid to these factors to facilitate earlier identification of high-risk patients and enable more targeted interventions. However, the current evidence remains limited. Before prediction models can be recommended for clinical application, more rigorously designed, transparently reported, and externally validated models are needed. In addition, as most existing studies were conducted in Chinese populations, the generalizability of these models to other countries and populations remains uncertain. Future studies should focus on developing and validating comparable prediction models in other populations or countries, strictly adhering to the PROBAST tool and the TRIPOD statement throughout model development and validation, in order to construct malnutrition risk prediction models for dialysis patients that feature lower risk of bias, robust design, standardized reporting, and external validation.

## Supplementary Material

Supplementary material.docx

## Data Availability

The data that support the findings of this study are available from the corresponding author upon reasonable request.
