# Supplementary material for: Risk prediction models for malnutrition in dialysis patients in China: a systematic review and meta-analysis
Source: Ren Fail. 2026 Jun 18;48(1):2687920. doi: 10.1080/0886022X.2026.2687920 (PMC13288544; doi:10.1080/0886022X.2026.2687920)

**Supplementary material**

[Table S1: Search strategy 1](#_Toc945010317)

[Table S2: GRADE evidence profile for area under the curve (AUC) 5](#_Toc2146828254)

[Table S3: Baseline characteristics of predictive factors in the included studies 6](#_Toc1962989665)

[Figure S1: Subgroup analyses exploring sources of heterogeneity in the prevalence of malnutrition among dialysis patients](#_Toc1769506424)

[7](#_Toc1769506424)

[Figure S2: Forest plot of predictors of the model 8](#_Toc116856366)

[Figure S3: Sources of heterogeneity for the predictor 9](#_Toc1962989665)

[Figure S4: Funnel plot to assess publication bias 1](#_Toc1464572839)0

[Figure S5: A trim-and-fill analysis 1](#_Toc618143159)1

**Table S1:** Search strategy

Search date: 2026.1.26

Search strategy for PubMed

| #4 | #1 AND #2 AND #3 | 1,473 |
| --- | --- | --- |
| #3 | (prediction model OR risk assessment OR risk scor* OR risk prediction OR predictive value )) | 2,346,184 |
| #2 | (( Malnutrition[Mesh] OR Protein-Energy Malnutrition[Mesh] OR Nutritional Deficiency OR Nutritional Deficiencies OR Undernutrition OR Malnourishment OR Malnourishments OR Malnutrition, Protein-Energy OR Malnutritions, Protein-Energy OR Protein Energy Malnutrition OR Protein-Calorie Malnutrition OR Malnutrition, Protein-Calorie OR Protein Calorie Malnutrition OR Marasmus OR Protein-Energy Wasting) | 213,689 |
| #1 | (Renal Dialysis[Mesh] OR Dialyses, Renal OR Renal Dialyse OR Dialysis, Renal Hemodialysis OR Hemodialyses Dialysis, Extracorporeal OR Dialyses, Extracorporeal OR Extracorporeal Dialyses OR Extracorporeal Dialysis OR Dialyses OR Dialyses, Peritoneal OR Dialysis, Peritoneal OR Peritoneal Dialyses) | 235,106 |

Search strategy for Embase

| #8 | #5 AND #6 AND #7 | 750 |
| --- | --- | --- |
| #7 | ('model':ab,ti OR 'prediction model':ab,ti OR 'risk scor*':ab,ti OR 'risk assessment':ab,ti OR 'risk prediction':ab,ti OR 'predictive value':ab,ti) | 4,507,224 |
| #6 | dialys*:ti,ab,kw OR hemodialys*:ti,ab,kw OR 'renal dialysis':ti,ab,kw OR 'peritoneal dialys*':ti,ab,kw | 298,706 |
| #5 | #1 OR #2 OR #3 OR #4 | 273,480 |
| #4 | 'Protein-Energy Wasting' OR 'energy protein malnutrition' OR 'malnutrition, protein energy' OR 'protein caloric deficiency' OR 'protein caloric malnutrition' OR 'protein calorie deficiency' OR 'protein energy malnutrition' OR 'protein-calorie malnutrition' OR 'protein-energy malnutrition' OR 'protein calorie malnutrition' | 10,795 |
| #3 | 'protein calorie malnutrition'/exp | 7,017 |
| #2 | 'deficient nutrition' OR 'malnourishment' OR 'severe acute malnutrition' OR 'underfeeding' OR 'undernourishment' OR 'undernutrition' OR 'malnutrition' | 272,782 |
| #1 | 'malnutrition'/exp | 243,040 |

Search strategy for Cochrane Library

| #1 | MeSH descriptor: [Malnutrition] explode all trees | 6,279 |
| --- | --- | --- |
| #2 | (Protein-Energy Wasting OR Undernutrition OR Malnourishment OR Malnourishments OR Nutritional Deficiencies OR Nutritional Deficiency) | 6,626 |
| #3 | #1 OR #2 | 11,225 |
| #4 | MeSH descriptor: [Dialysis] explode all trees | 302 |
|  | (Dialyses OR renal dialysis OR Peritoneal Dialysis OR Hemodialysis OR Peritoneal Dialyses OR Hemodialyses):ti,ab,kw | 27,586 |
| #5 | (prediction model):ti,ab,kw OR (risk score):ti,ab,kw OR (risk assessment):ti,ab,kw OR (risk prediction):ti,ab,kw OR (predictive value):ti,ab,kw | 125,838 |
| #6 | #3 AND #4 AND #5 AND #6 | 95 |

Search strategy for Web of Science

| #4 | #4 AND #3 AND #2 AND #1 | 779 |
| --- | --- | --- |
| #3 | (TS=(risk prediction OR risk assessment OR prediction model OR predictive value) | 1,754,118 |
| #2 | (TS=(Malnutrition* OR Undernutrition* OR Protein-Energy Wasting OR Protein-Energy Malnutrition OR Protein Energy Malnutrition OR Protein-Calorie Malnutrition OR protein calorie malnutrition OR Nutritional Deficiency OR Nutritional Deficiencies ) | 82,332 |
| #1 | (TS=(dialys* OR hemodialys* OR renal dialysis OR peritoneal dialys*)) | 179,120 |

Search strategy for China National Knowledge Infrastucture (CNKI)

| #1 | 透析 + 血液透析 + 维持性血液透析 + 腹膜透析 + 肾透析 + 终末期肾病 | 136,836 |
| --- | --- | --- |
| #2 | 营养不良 + 蛋白质能量消耗 + 蛋白质能量营养不良 + 蛋白质能量摄入不足 + 蛋白质能量营养不足+营养风险 | 40,659 |
| #3 | 预测 + 风险预测模型+预测模型 + 模型构建 + 模型验证 + 列线图+机器学习+风险预测 | 1,406,432 |
| #4 | #1 AND #2 AND #3 | 170 |

Search strategy for Wanfang Database

| #1 | 透析 OR 血液透析 OR 维持性血液透析 OR 腹膜透析 OR 肾透析 OR 终末期肾病 | 186,309 |
| --- | --- | --- |
| #2 | 营养不良 OR 蛋白质能量消耗 OR 蛋白质能量营养不良 OR 蛋白质能量摄入不足 OR 蛋白质能量营养不足 OR 营养风险 | 53,984 |
| #3 | 预测 OR 风险预测模型 OR 预测模型 OR 模型构建 OR 模型验证 OR 列线图 OR 机器学习 OR 风险预测 | 1,427,956 |
| #4 | #1 AND #2 AND #3 | 97 |

Search strategy for China Science and Technology Journal Database (CQVIP)

| #1 | 透析 OR 血液透析 OR 维持性血液透析 OR 腹膜透析 OR 肾透析 OR 终末期肾病 | 129,266 |
| --- | --- | --- |
| #2 | 营养不良 OR 蛋白质能量消耗 OR 蛋白质能量营养不良 OR 蛋白质能量摄入不足 OR 蛋白质能量营养不足 OR 营养风险 | 53,464 |
| #3 | 预测 OR 风险预测模型 OR 预测模型 OR 模型构建 OR 模型验证 OR 列线图 OR 机器学习 OR 风险预测 | 1,762,1022 |
| #4 | #1 AND #2 AND #3 | 206 |

Search strategy for Chinese Biomedical Literature Service System (SinoMed)

| #1 | ( "透析"[常用字段:智能] OR "血液透析"[常用字段:智能] OR "维持性血液透析"[常用字段:智能] OR "腹膜透析"[常用字段:智能] OR "肾透析"[常用字段:智能] OR "终末期肾病"[常用字段:智能]) | 105,517 |
| --- | --- | --- |
| #2 | ( "营养不良"[常用字段:智能] OR "蛋白质能量消耗"[常用字段:智能] OR "蛋白质能量营养不良"[常用字段:智能] OR "蛋白质能量摄入不足"[常用字段:智能] OR "蛋白质能量营养不足"[常用字段:智能] OR "营养风险"[常用字段:智能] OR "蛋白质能量浪费"[常用字段:智能]) | 68,193 |
| #3 | ( "预测"[常用字段:智能] OR "风险预测模型"[常用字段:智能] OR "预测模型"[常用字段:智能] OR "模型构建"[常用字段:智能] OR "模型验证"[常用字段:智能] OR "列线图"[常用字段:智能] OR "机器学习"[常用字段:智能] OR "风险预测"[常用字段:智能]) | 410,033 |
| #4 | #1 AND #2 AND #3 | 219 |

Search strategy for CINAHL

| #1 | SU (Dialyses OR renal dialysis OR Peritoneal Dialysis OR Hemodialysis OR Peritoneal Dialyses OR Hemodialyses) | 187,806 |
| --- | --- | --- |
| #2 | SU (Malnutrition OR Protein-Energy Wasting OR Undernutrition OR Malnourishment OR Malnourishments OR Nutritional Deficiencies OR Nutritional Deficiency) | 74,700 |
| #3 | SU (prediction model OR risk score OR risk assessment OR risk prediction OR predictive value) | 1,194,483 |
| #4 | #1 AND #2 AND #3 | 197 |

**Table S2: GRADE evidence profile for area under the curve (AUC)**

|  | Certainty assessment | | | | | | |  |  |
| --- | --- | --- | --- | --- | --- | --- | --- | --- | --- |
| No of  studies | Study design | Risk of  bias | Inconsistency | Indirectness | Imprecision | Publication bias | Other  considerations | outcome | Quality of evidence |
| GRADE evidence profile for area under the curve (AUC). | | | | | | | | | |
| 9 | observational studies | not  serious ^a^ | not serious ^b^ | serious ^c^ | not serious ^d^ | Undetected | none | Pooled effect  AUC = 0.83  (95% CI 0.76- 0.90) | ⨁⨁⊙⨁ MODERATE |

**Explanations**

a. All of the pooled estimate weight comes from studies with low risk of bias (GRADE guidelines: 4. Rating the quality of evidenced study limitations (risk of bias)

b. Despite a high I² value, a predefined subgroup analysis was conducted, and the heterogeneity has been reasonably explained..

c. Differences in outcome definition.

d. The majority of pooled estimate weight comes from data with no significant evidence of such bias.

**[Table S3: Baseline characteristics of predictive factors in the included studies](#_Toc1962989665)**

| Predict Factors | Study | Malnutrition | Non-malnutrition |
| --- | --- | --- | --- |
| Serum calcium  (mmol/L) | Weina Wang (2023) [27] | 2.22 ± 0.21 | 2.27 ± 0.23 |
|  | Ziwei Mei (2022) [28] | 2.22 (2.09,2.34) | 2.33 (2.20,2.41) |
|  | Ziwei Mei (2023) [12] | 2.23 ± 0.21 | 2.30 ± 0.22 |
| Triglycerides  (mmol/L) | Weina Wang (2023) [27] | 1.33 (0.94,2.00) | 1.86 (1.26,2.66) |
|  | Si Chen (2022) [29] | 1.39 (0.90∼2.11) | 1.90 (1.26∼2.74) |
|  | Ziwei Mei (2023) [12] | 1.39 (0.98, 1.95) | 1.78 (1.17, 2.53) |
| Vitamin D  (ng/mL) | Si Chen (2022) [29] | 15.04 (11.00~31.56) | 25.91 (13.77~36.99) |
|  | Mingmei Ding (2024) [31] | 18.2 (13.5,25.6) | 25.6 (14.6,36.4) |
| NT-proBNP  (ng/L) | Si Chen (2022) [29] | 3971.50 (2275.50,16426.00) | 3074.00 (1817.25,6316.50) |
|  | Mingmei Ding (2024) [31] | 4112 (2564,6549) | 3756 (2012,5145) |
| Male sex, n(%) | Si Chen (2022) [29] | 98 (56.00) | 141 (68.78) |
| Male/Female  (n) | Mingmei Ding (2024) [31] | 60/65 | 122/66 |
| Comorbid diabetes  (n) | Guoting Ma (2023) [26] | 116 | 242 |
|  | Mingmei Ding (2024) [31] | 67 | 59 |
| Kt/V (n) | Guoting Ma (2023) [26] | ≥1. 2: 189  < 1. 2: 136 | ≥1. 2: 723  < 1. 2: 307 |
|  | Xueqin Liu (2022)[13] | ≥1. 2: 186  < 1. 2: 134 | |
| Kt/V, n(%) | Yingying Zhang (2025) [33] | ≥1. 2: 24 (35.82)  < 1. 2: 43 (64.18) | ≥1. 2: 9 (15.25)  < 1. 2: 50 (84.75) |
|  | Min Wei(2023) [25] | ≥1. 2: 26 ( 31. 33)  < 1. 2: 57 ( 68. 67) | ≥1.2: 47 ( 67. 14)  < 1. 2: 23 ( 32. 86) |
| Age (n) | Guoting Ma (2023) [26] | ≥60: 153  <60: 172 | ≥60: 349  <60: 681 |
|  | Xueqin Liu (2022) [13] | ≥60: 142  <60: 178 | |
| Age (years) | Min Wei(2023) [25] | 1. 95±12. 84 | 52. 94±14. 23 |
|  | Weina Wang (2023) [27] | 56.37±14.16 | 53.49±13.29 |
|  | Ziwei Mei (2023) [12] | 59.31±11.74 | 55.58±10.85 |
|  | Ziwei Mei (2022) [28] | 61.00±10.43 | 55.44±10.99 |
| Age, n(%) | Yingying Zhang (2025) [33] | ≥60: 40 (59.70)  <60: 27 (40.30) | ≥60: 20 (33.90)  <60: 39 (66.10) |

Note: All differences presented are statistically significant (P < 0.05). Mean ± SD is presented for variables according

to the normal distribution, while median (IQR) is presented for variables with the abnormal distribution.

**Figure S1: Subgroup analyses exploring sources of heterogeneity in the prevalence of malnutrition among dialysis patients.**


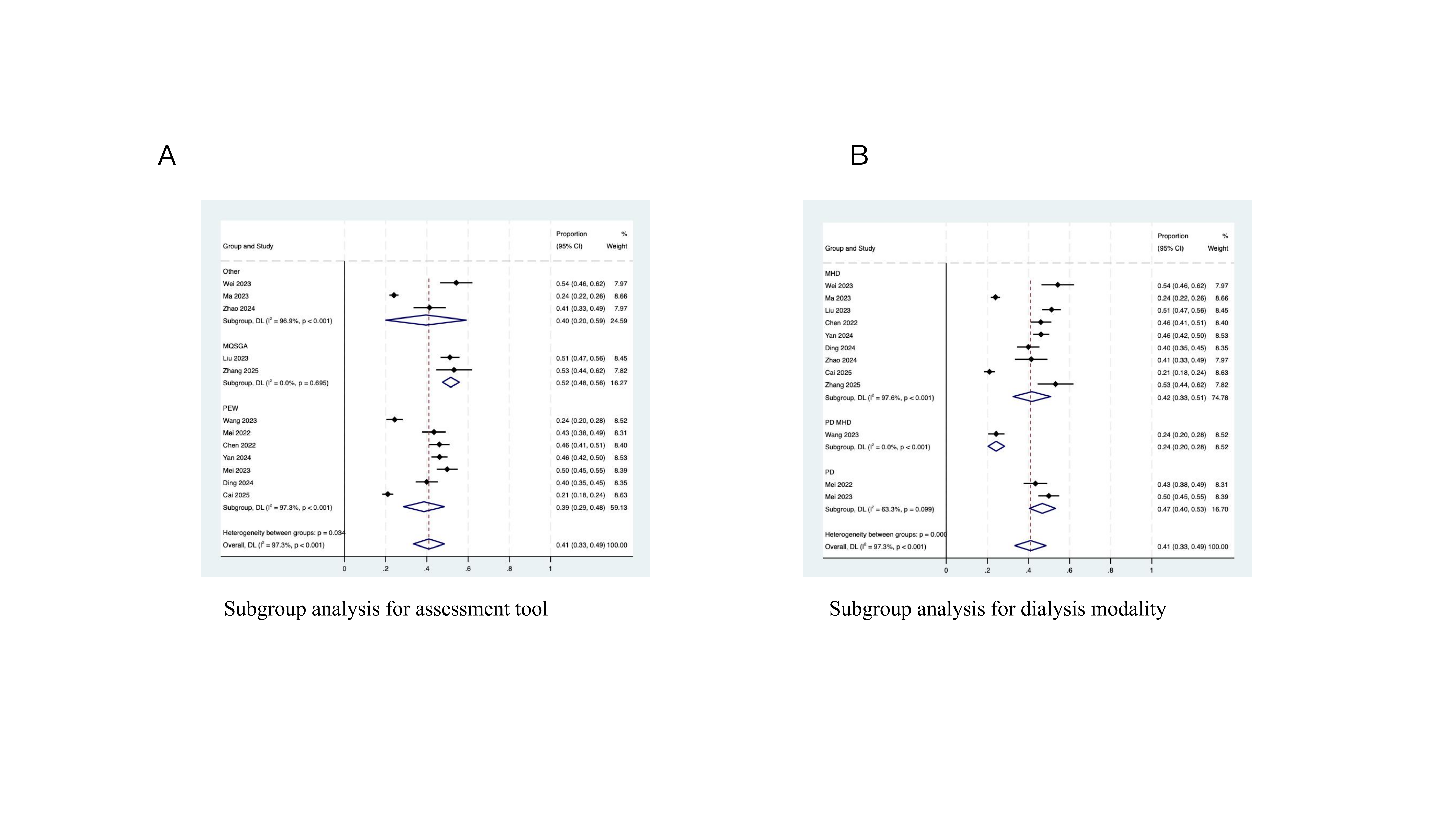


**Figure S2: Forest plot of predictors of the model**


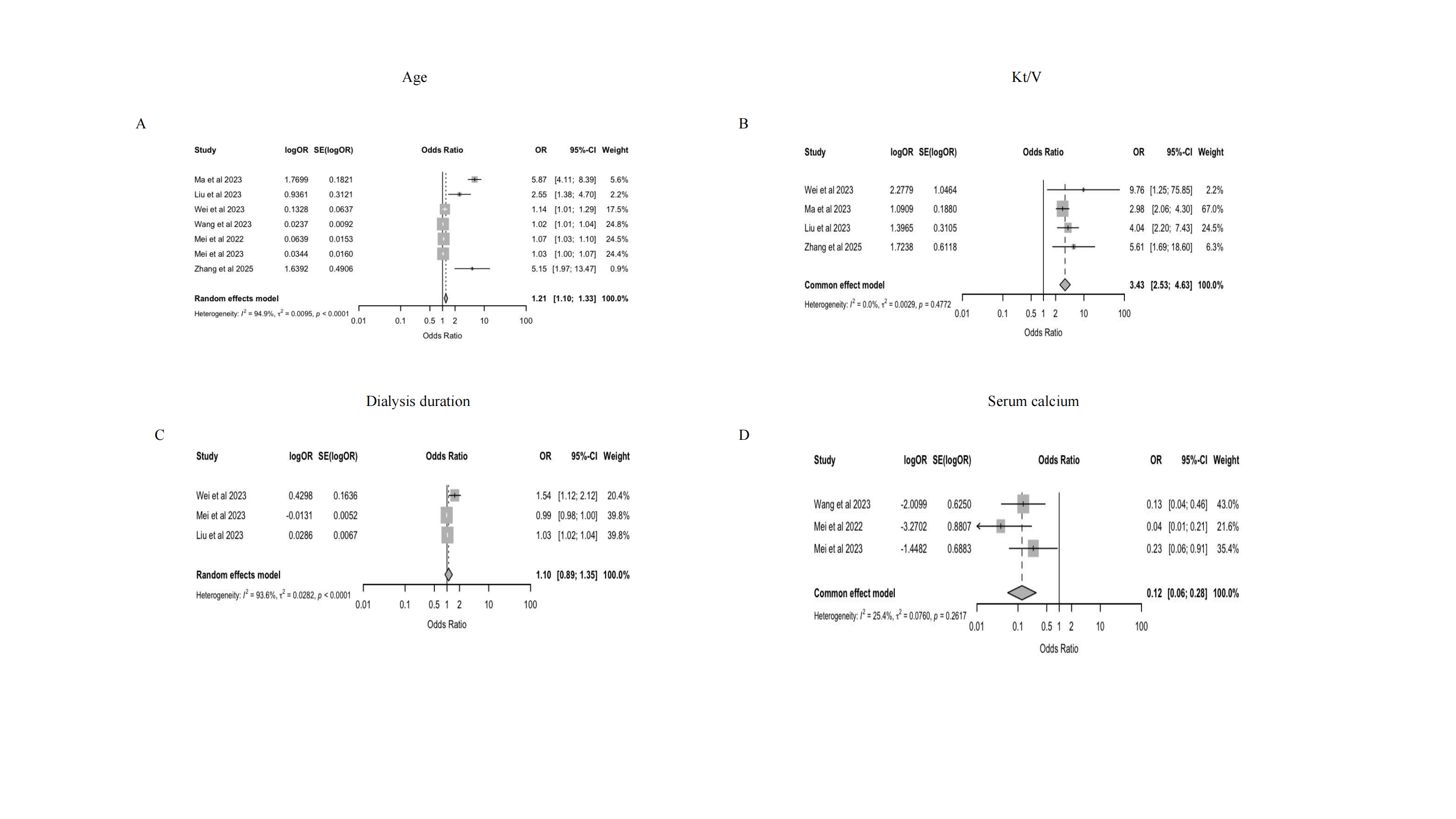


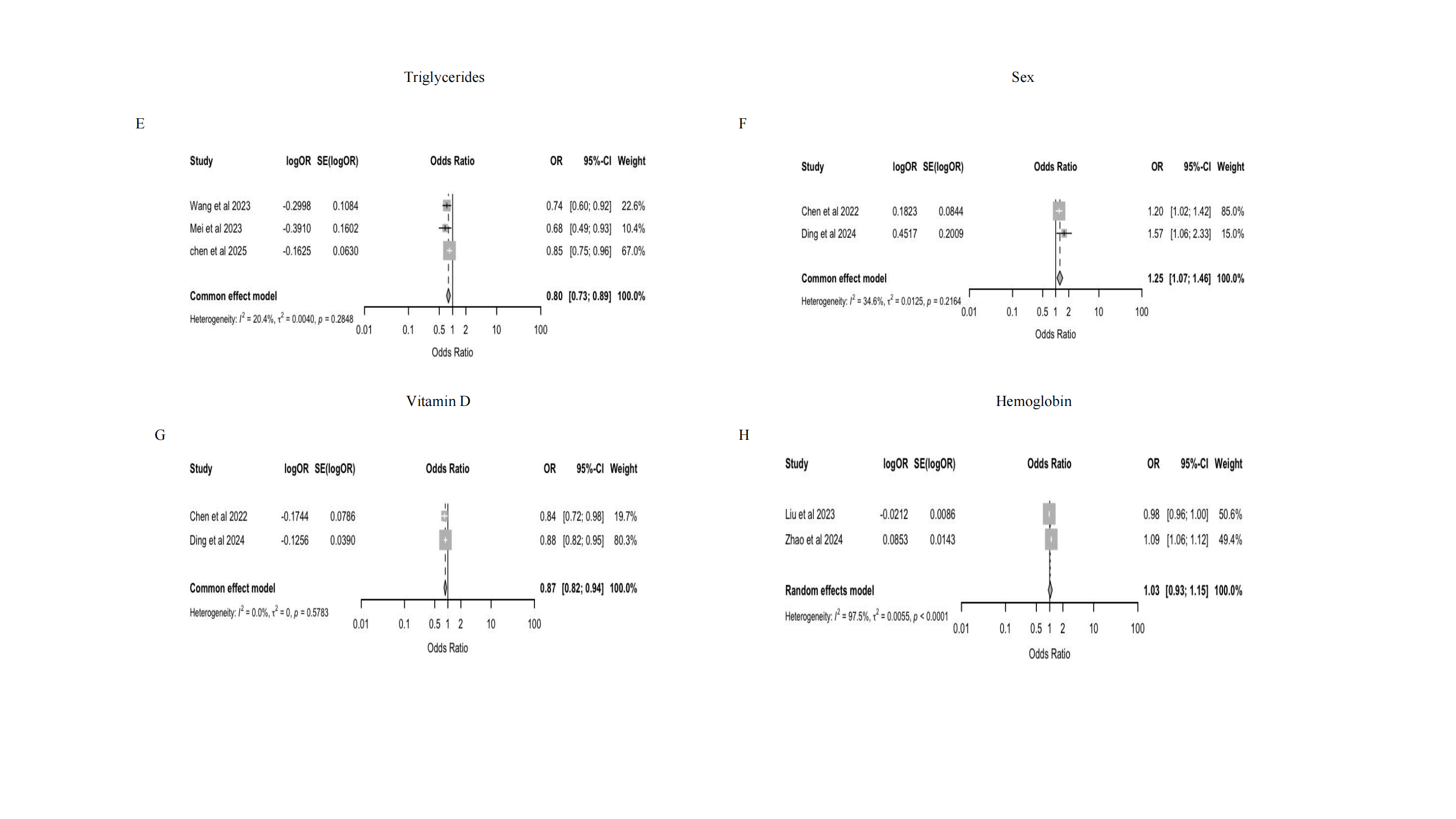


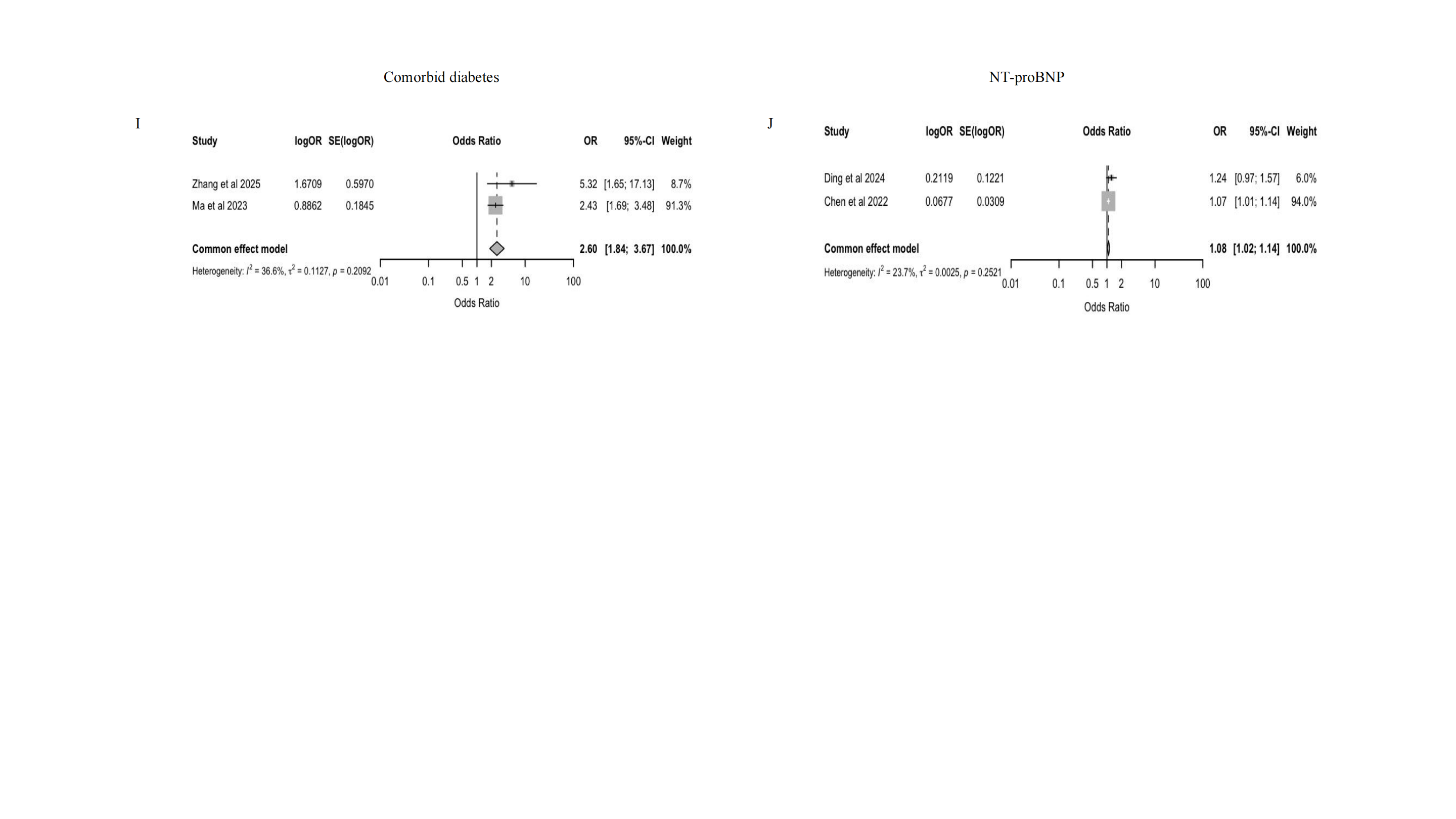


**Figure S3: Sources of heterogeneity for the predictor**

**
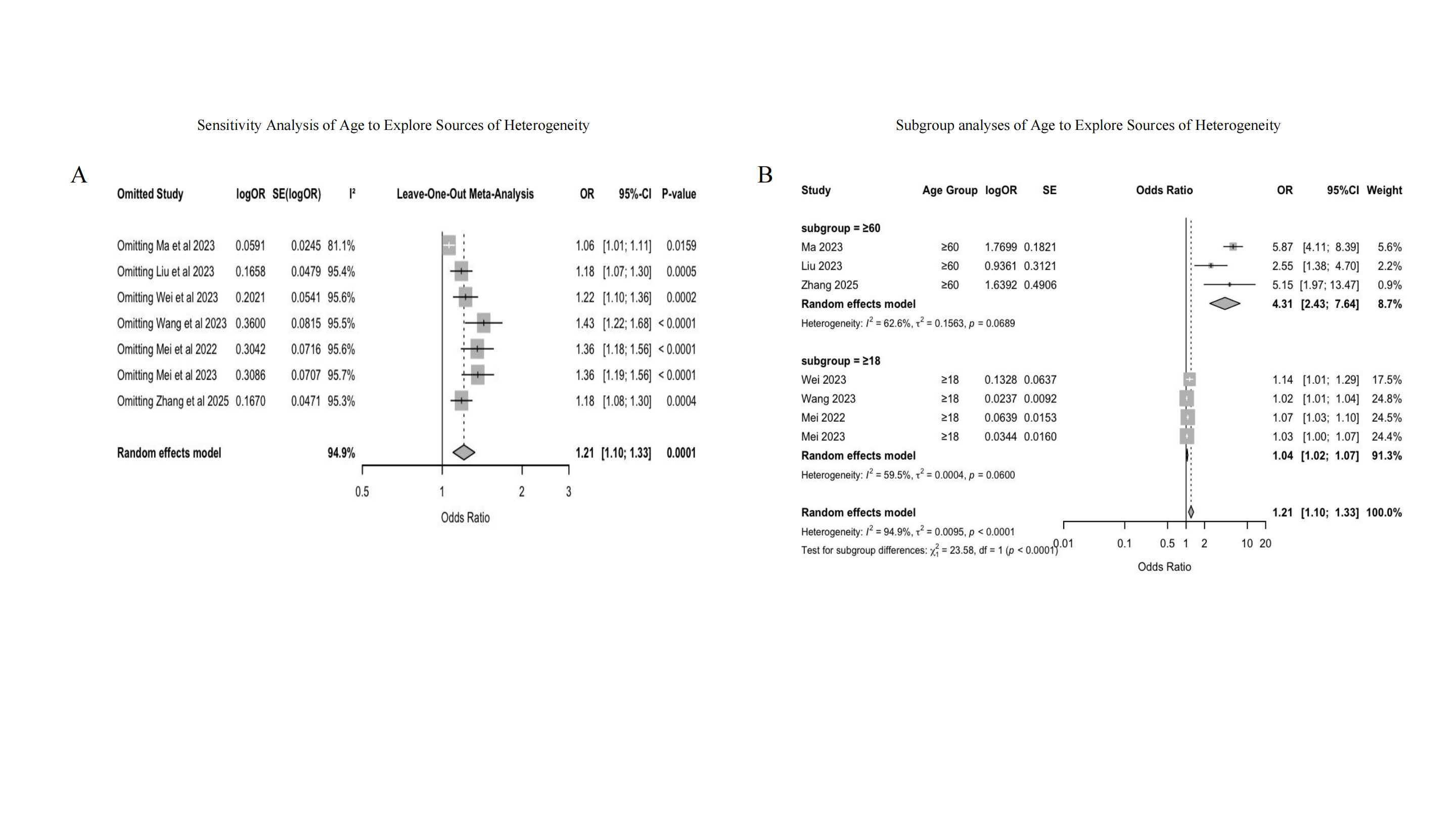
**

**Figure S4: Funnel plot to assess publication bias**


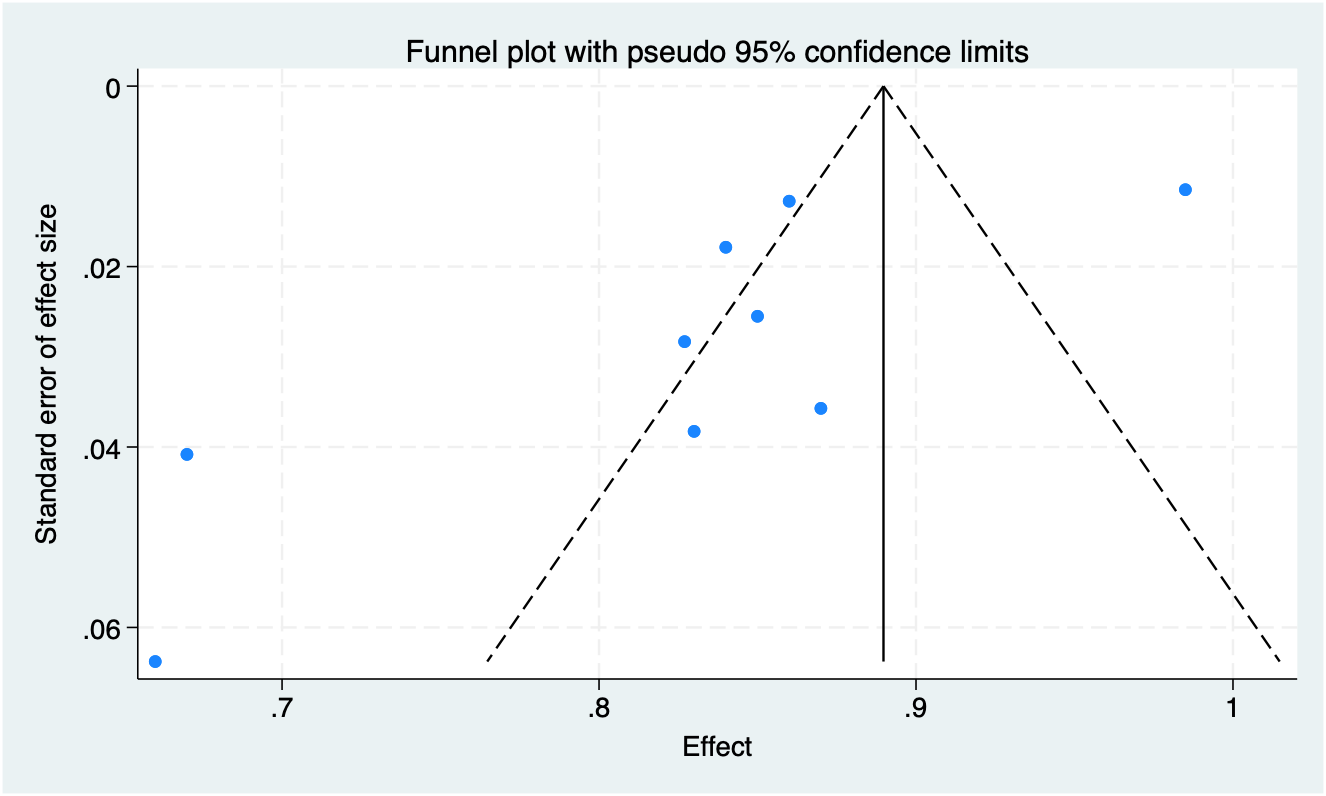


**Figure S5: A trim-and-fill analysis**


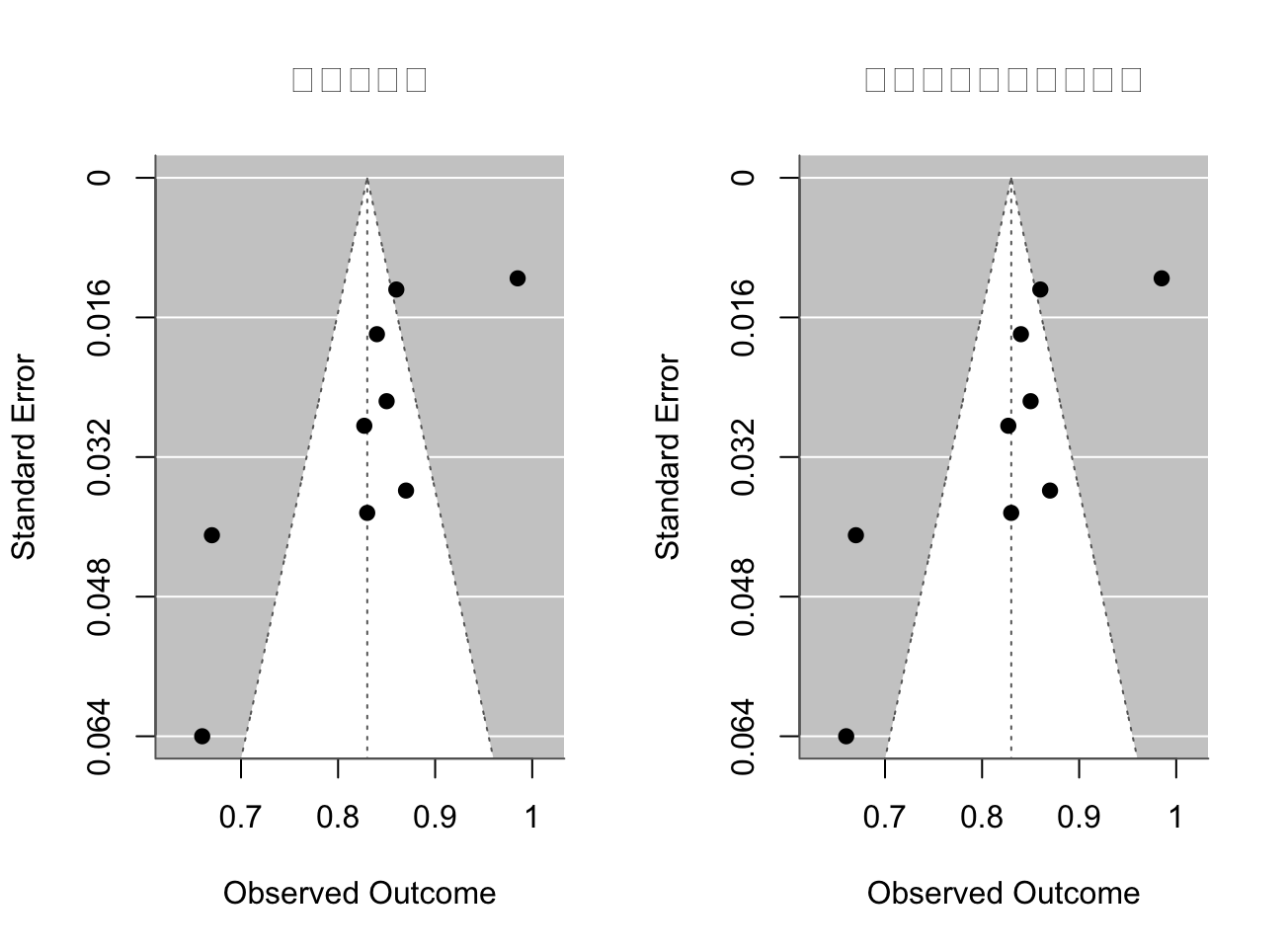

Supplement: Supplementary material.docx [file IRNF_A_2687920_SM9321.docx]
